# Study protocol: Exploring the use of Family Health Histories in the African American community to reduce health disparities in Flint, Michigan

**DOI:** 10.21203/rs.3.rs-4131949/v1

**Published:** 2024-04-01

**Authors:** Kent D. Key, Lena Lewis, Courtney Blanchard, Alla Sikorskii, Minal Patel, Todd Lucas, Tabia Henry Akintobi, Sarah Bailey, E. Hill Loney, Jennifer E. Johnson

**Affiliations:** Michigan State University College of Human Medicine, Lansing, MI, United States; Michigan State University College of Human Medicine, Lansing, MI, United States; Michigan State University College of Human Medicine, Lansing, MI, United States; Michigan State University College of Human Medicine, Lansing, MI, United States; University of Michigan–Ann Arbor; Michigan State University College of Human Medicine, Lansing, MI, United States; Morehouse School of Medicine, Atlanta, GA, United States; Bridges into the Future, Flint, MI, United States; Community Based Organization Partners, Flint, MI, United States; Michigan State University College of Human Medicine, Lansing, MI, United States

**Keywords:** African Americans, Health Disparities, Family Health History, Community Based Participatory Research, Randomized Trial

## Abstract

**Background:**

Health disparities are costly and preventable differences in disease progression that disproportionately affect minority communities such as African Americans. Practices to reduce health disparities can be rooted in prevention, particularly through screening tools. Family Health History tools are preventative screening mechanisms meant to explore family history to better understand how an individual’s health can potentially be predicted or impacted. These tools are underutilized in the African American community. Contributions to this underutilization include a lack of cultural tailoring in the tools, a lack of health literacy in community members, and a lack of effective health communication. The Family Health History Study will create a culturally appropriate Family Health History toolkit to increase family health history utilization and ultimately decrease health disparities.

**Methods:**

The proposed sample will be composed of 195 African American adults ages 18 + who live in Genesee County, Michigan. The study consists of two phases: the development phase and the randomized pilot study phase. The goal of the development phase (n = 95) is to explore how Family Health History toolkits can be modified to better serve the African American community using a community based participatory research approach and to create a culturally tailored family health history toolkit. In the pilot study phase, 100 participants will be randomized to the culturally tailored toolkit or the current standard Family Health History toolkit. Outcomes will include feasibility and acceptability of the intervention.

**Discussion:**

This study will result in a culturally appropriate Family Health History tool that is co-developed with community members that can be utilized by African American adults to better understand their family health histories.

**Trial registration:**

Clinicaltrials.gov: NCT05358964 Date: May 5, 2022

## Background

Health disparities (HD) are preventable differences in disease progression and outcomes that disproportionately impact certain populations. HD are costly and preventable. Although many factors impact HD, racism is especially significant. In 2018, the economic burden of racial and ethnic health inequities in the United States was $450.8 billion.”^75^ African Americans (AAs) suffer disproportionately across most diseases compared to Whites.^2,22^ AAs have the highest death rate and shortest survival of any racial and ethnic group in the US across most types of cancers and other preventable diseases.^3^ The most prevalent common health disparity diseases in the AA community include diabetes, heart disease, high blood pressure, stroke, HIV, STDs/STIs, cancer, and cardiovascular disease, most of which can be prevented.^1,2^ More effective strategies for reducing the disproportionate burden that HD have on the AA community are needed. To reduce the disparities that exist, research must explore tools, resources, and skills that mitigate the negative consequences of HD and ultimately work to eliminate them entirely.

Current practices to address HD include preventative behaviors such screenings that inform proactive measures to prevent disease.^1–3^ Family Health History (FHH) tools are an effective way to promote screening and early detection, potentially preventing the onset of HD-affected diseases.^2,23^ FHH tools provide a structured way for individuals to identify and catalog the social, genetic, and environmental factors contributing to their own disease risk by collecting, recording, and sharing family health information.^23,24^ Family health, which reflects both inherited and acquired environmental factors, is one of the most important risk factors for many health disparity conditions including cancer, heart disease, cardiovascular disease, hypertension, diabetes and other chronic conditions.^25–28^

National and international scholars alike recognize the roles that FHHs plays in supporting tailored disease prevention.^29^ Understanding one’s FHH can motivate lifestyle changes, influence clinical interventions, enhance individual empowerment, and help to personalize medical care.^23,30^ National efforts, like those undertaken by the Pittsburg’s Center for Minority Health, recognize and suggest that one key mechanism in reducing HD in AA and other minority communities is to use FHHs.^2^ Although many FHH toolkits have been created to assist families in gathering FHH information, these tools, due to their design or the lens of the developer, tend to be catered to the general population, not accounting for the cultural and ethnic nuances, communication preferences, and health literacy levels of the AA community.^4^ A randomized trial showed that FHH tools were effective among AA, but recommended that the tools be adapted and tailored for AA communities to 1) appeal to the AA culture; 2) take into consideration the literacy levels of the AA community; and 3) increase understanding of the utility of FHH.^1^

**Figure 1:**
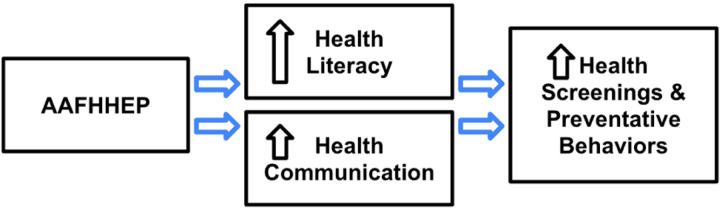
Study Model

Failure to effectively engage AA in the conception and creation of culturally relevant FHH tools and activities, along with structural and cultural barriers, likely contributes to their underutilization in this population.^4^ This negatively impacts screening and preventative measures that could prevent the onset of disease, illness, and ultimately death. Underutilization of FHH tools in the AA community may also be due to a general lack of health literacy and effective health communication.^1,31^ AA have lower health literacy than European Americans,^35^ and health literacy remains a critical issue in the AA community. Wang et al. (2010), systematically identified and evaluated the readability of current FHH tools and found that the majority of FHH tools were written at an 8th grade reading level.^36^ They concluded that the tools were difficult to read and comprehend by most people, and that this compromises the potential impact the FHH can have on a population level.^36^ Given this contect, this study uses a modified version of the Baker’s Measuring Health Literacy Model (Fig. 1). This model posits that improved health outcomes will result from the intersection of health literacy and the ability to orally communicate health messages to others (health communication).

The goal of this study is to create a culturally tailored FHH tool, co-developed by members of the AA community to inform, educate, and empower AAs about health issues related to their family genealogy. Applying the knowledge gained via FHHs to increase preventative behaviors, including screenings, can link people to needed health services to prevent the onset of disease and illness. The Family Health History Study will develop and pilot test a culturally appropriate FHH toolkit to increase the understanding, utilization, and uptake of FHHs in the AA community, increase health literacy, and increase effective health communication. The ultimate goal is to help address HD and advance health equity.

This study uses a Community Based Participatory Research (CBPR) process. CBPR is a partnership approach to research that equitably involves community members, organizational representatives, researchers, and others in all aspects of the research process, with all partners in the process contributing expertise and sharing in decision-making. CBPR emphasizes a shared frame of equity as it relates to power and benefits of research for both the researcher and the participant/subject.^37,38^ Unlike traditional positivist science, research results are contextualized within specific community settings and the participants should play an equitable role in the generation of that research context.^38^ The CBPR approach is rooted in equity and social justice, which is particularly important in the AA community and other communities of color.^39,40^ The National Institute on Minority Health and Health Disparities embraces CBPR approaches to engaging communities in research to maximize research translation and enhance the effectiveness of interventions to improve health outcomes.^41^ This further emphasizes the necessity of developing a CBPR created, culturally appropriate FHH prevention toolkit.

## Methods and Study Design

### Project Overview

The Family Health History Study is a longitudinal study with AA adults composed of two phases: the development phase and pilot study phase. During the development phase of the study, participants will engage in focus groups, workshops, and an open trial. In the randomized pilot study phase, the African American Family Health History Education Program (AAFHHEP; the intervention group) will be compared to an Existing, non-tailored Family Health History Education Program (EFHHEP; the control group). In this project, assessment of feasibility and acceptability of the intervention and research procedures is the ultimate analytical goal.

### Participants

#### Inclusion and Exclusion Criteria

Individuals who self-identify as AA, are 18 + years old, live in Genesee County, Michigan, and speak English are eligible to participate in the study. Development phase participants include 40 AA in focus groups, 40 AA in workshops and 15 AA in the open trial. In the randomized pilot study phase, 100 AA individuals will be recruited over the course of 7 months. In total, 195 AAs will participate across all components of the study.

#### Recruitment and Enrollment

Participant recruitment strategies will include flyers, public service announcements, and partnerships with the Community Based Organization Partners (CBOP), the Genesee County Health Department (GCHD), and other Michigan State University-based community councils, action boards, resident consortiums, and advisory groups. These methods were already conducted for the development phase and will continue for the pilot study phase. Potential participants will reach out directly to the study via phone and a research assistant will contact them. Once the person arrives to the scheduled focus group, workshop, open trial session, or randomized control trial session, they will be consented into the study by a research assistant.

#### Procedures:

**Figure 2:**
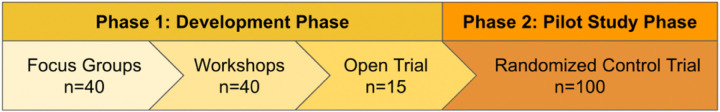
Study Phases and Components

This five-year study includes 4 components: focus groups, workshops, open trial, and a pilot randomized controlled trial (RCT). Figure 2 details the overall timeline of the study, with focus groups, workshops, and the open trial being performed consecutively during the development phase and the RCT being conducted during the pilot study phase.

Focus groups will be conducted during the first year of the study. Four focus groups composed of 8–12 people will be formed for a total of 40 AA participants. The purpose of the focus groups is to describe perspectives on inherited health and approaches to family health communication. The discussions will be audiotaped, and a research assistant will be appointed as a note taker. Transcriptionists will transcribe the tapes and Kent Key, PhD (study principal investigator) will perform qualitative analyses on the data to inform workgroup conceptualization and creation of the AAFHHEP.

The next part of the development phase will consist of four workgroups with 8–12 participants in each one, totaling 40 AA participants. The workgroups will consist of AA community members, the research team, the Flint & Genesee County Literacy Network, and local healthcare providers. Themes from the focus groups will be incorporated into the workgroups to co-develop and tailor a culturally appropriate FHH intervention tool for AA. Participants will review current FHH tools to identify culturally appropriate core sections for a new FHH. Next, they will identify and create language and visuals they deem community appropriate for incorporation into the AAFHHEP. Finally, participants will work with the Flint/Genesee Literacy Network to review developed materials for cultural appropriateness and health literacy required to understand the FHH.

The final part of the development phase is the open trial. This component consists of 15 AA adults that meet the aforementioned eligibility criteria. The open trial provides the opportunity to refine the created FHH toolkit. Study participants will fill out a session evaluation form asking them about their opinions of each session and how sessions could be made more helpful. Participants will also complete an intervention-specific End-of-Intervention Questionnaire, which addresses their perceptions of the helpfulness of each intervention component and their comfort with research procedures such as audiotaping and timing of the two FHH sessions. Participants will discuss their responses at an exit interview with the study principal investigator to aid in manual revisions.

The pilot study phase consists of the RCT. During the RCT, the intervention group will receive the AAFHHEP while the control group receives the EFHHEP. A total of 100 participants over the course of seven months will be recruited for this portion of the study. AAFHHEP participants will receive the intervention in two 60–90-minute sessions about 2 weeks apart. Session 1 focuses on understanding and reviewing FHH tools and its utility in risk assessment, including the AAFHHEP toolkit. Session 2 addresses family engagement strategies and provider engagement strategies. This session helps participants to develop personalized engagement plan resulting to actively engage family and their primary care providers (PCPs) utilizing the AAFHHEP toolkit. This toolkit will tailor messages around culture and spirituality that may preclude individuals from communicating. Participants will discuss what an FHH is, how choices affect health, how genetics affect health, what information to collect, how to engage family members in the conversation, how to organize FHH, and how to share information with their PCP. The feasibility and acceptability of AAFHHEP and EFHHEP will be assessed by examining rates of treatment attendance, rates of treatment completion (attending both scheduled group sessions) and drop-out, and scores on the End of Treatment Questionnaire, described below.

### Measures

#### Demographic/screening measures:

All participants will begin the study by completing demographic information, including age, educational level, marital status, occupation, employment (status, # hours per week), income, and race. These measures will be assessed once at the baseline of each component. Additional measures will be assessed at various points during the study. Table 1 shows an overall timeline of the distribution of each measure.

#### Primary Outcome Measures:

Primary outcomes will be assessed at baseline, post three months, and post six months in all participants. A revised Client Satisfaction Questionnaire (CSQ-8-R) will be administered and contains questions investigating the quality of the intervention, satisfaction with the intervention, if the intervention has helped connect the participant with other resources, and more. A modified Genetic Alliance, “Does it Run in the Family” assessment will also be administered. This measure has items such as: “The toolkit made our conversations with family better; toolkit has encouraged me to talk to my own family about family health history.”^67^

#### Secondary Outcome Measures:

Secondary outcomes will also be assessed at baseline, post 3 months, and post 6 months in all participants. Comprehension of the FHH tool will be assessed using a subscale from the Functional Health Literacy Scale such as: print too small, content was difficult to read, etc. Number of screenings requested, and number of screenings received will both be assessed using a modification to the Family Health Communication Quotient (FHCQ). This measure will also be used to assess the number of screenings requested and received in the 6 months prior to baseline.

Health literacy will be assessed using subscales of the Health Literacy Questionnaire (HLQ) Communicative and Critical Health Literacy Scale (CCHL).^69^ Comprised of nine independent scales, the HLQ was modified to assess understanding of health information and use of that information to make health-related decisions.^69,70^ Health communication will be determined using the HLQ scale to assess the ability to actively engage providers and support (family) which assesses the ability to communicate thoughts about one’s health.^69,70^ The Family Health Communication Quotient (FHCQ) will be used as a secondary measure of health communication. The FHCQ measures a general orientation toward discussion about health between family members, frequency and effects of discussions, communication style when discussing health, and attitudes toward health practices.^68,71^ It will ultimately be used to analyze the length and meaningfulness of discussions with family members.

### Analytic Plan

#### Data Analysis:

To achieve the goal of understanding the assessment and feasibility of this intervention, study recruitment and refusal rates, participants’ willingness to be randomized, follow-up rates, reliability, range of responses to study questionnaires, and success of the interventionist training program will be examined. The feasibility and acceptability of AAFHHEP and EFHHEP, as described previously, will be summarized with descriptive statistics. The acceptability of both interventions using data from CSQ-8-R satisfaction questionnaire and detailed exit interviews will be examined. Internal consistency and interrater reliability of fidelity scales will be computed

Primary RCT analyses will be intent-to-treat (using data from all enrollees as randomized) for the outcomes of FHH use (FHH discussed with a family and health care provider). Secondary dose- response analyses within intervention group evaluating effects of receiving 0, 1, or 2 intervention sessions will also be conducted. Longitudinal data will be analyzed using linear mixed effects or generalized linear mixed effects models with the appropriate error distribution (e.g., Binary) and covariance adjustment for the baseline version of the outcome where possible. These models can accommodate data missing at random and will be used to estimate the effect sizes at 3 and 6-month time points.

#### Primary and Secondary Outcomes:

Odds ratios and 95% confidence intervals for discussing the FHH with family and discussing the FHH with a physician (separately) will be calculated. For discussing the FHH with family, exploratory tests for differences between conditions will use GLME, with baseline FHCQ scores that assess the family’s general orientation toward health discussions as a covariate. With regards to discussing the FHH with a physician, exploratory tests for differences between conditions will use GLME with binary errors with the baseline number of past 6-month discussions with a physician as a covariate. For participants who discussed the FHH with family, effect size (Cohen’s d) and 95% confidence intervals for self-reported length of FHH discussion with family and meaningfulness of discussion with family will be calculated.

To explore secondary outcome measures, we will use separate HLM analyses to calculate effect size (Cohen’s d) and 95% confidence intervals for satisfaction with and for understandability of the FHH intervention at Month 3 and Month 6. Effect sizes and 95% confidence intervals for arm differences in the number of screenings requested and number of screenings received across 3- and 6-month follow-ups will also be calculated. Effect sizes (Cohen’s d) and 95% confidence intervals for between-arm differences will be calculated for health literacy (HLQ scores) and for health communication (HLQ scores; FHCQ scores) at 3 and 6 months.

Although this intervention development study is not formally powered to test mediation, Preacher and Hayes approach^74^ will be used to explore whether health literacy and/or health communication appear to be reasonable to test as mechanisms of the effects of the culturally adapted FHH (relative to the standard FHH) on number of health screenings received, in a larger trial. Sex and age as potential moderators of intervention effects will be explored.

#### Sample Size Adequacy:

The primary purpose of this pilot study is to develop the intervention and test feasibility/acceptability of interventions and research procedures as well as preliminary efficacy of the intervention. In the RCT, with follow-up data from 80 participants in intent to treat analyses (about 40 per condition, after the projected 20% attrition before month 3), we will have statistical power adequate (> = .80) to detect medium to large effects (Cohen’s d = .63 for continuous outcomes; 80% vs. 50% for dichotomous outcomes) with alpha of .05. The primary emphasis will be on estimating the effect for differences between conditions.

## Discussion

The Family Health Histories Study seeks to create a culturally appropriate FHH tool to inform AAs about their health and the health of their families with the intention of increasing preventative behaviors. Preventative behaviors will lead to improved health and reduce incidence of disease and illness. This is the first study to co-create and co-design and AA specific FHH tool. The community-engaged study design allows for incorporation of opinions from those who will benefit from the FHH directly. The setting of the study in Genesee county, MI is especially meaningful given the historic mistreatment of the county’s population, including the ongoing Flint water crisis. The inclusion of focus groups, workshops, and an open trial prior to conducting an RCT creates iterative opportunities to identify issues, implement changes, and improve the tailored FHH tool to make it more culturally relevant. Conducting follow ups with participants of the RCT at three and six months allows for assessment of immediate and longer-term outcomes. Assessments also include potential mediators of intervention effects, including health literacy and health communication, to inform a subsequent fully-powered trial.

Results from this study can inform how primary care provider’s structure their family health history forms, particularly when working with marginalized communities and given health literacy concerns. Meaningful FHH discussions between patients and providers can further contribute to addressing HD and increasing preventative health behaviors. Future interventions guided by this research include expanding the intervention guide to geographies beyond Genesee County, MI. Geographical areas with a large AA community will provide a greater understanding of the generalizability of the culturally tailored tool.

## Conclusion

HD are caused by a multitude of factors and, as a result, require interventions on multiple levels. While efforts are being made to mitigate these effects, continued research is needed to explore additional mechanisms through which HD operate and to identify ways to combat them. This study will create a culturally tailored AA FHH tool, lead to subsequent testing of its effects on healthcare utilization and outcomes, with the goal of reducing HD.

## Abbreviations

AA: African American
HD: Health Disparities
AAFHHEP: African American Family Health History Education Program
EFHHEP: Existing, non-tailored Family Health History Education Program
